# Sodium-glucose transporter 2 inhibitors and cardiovascular-kidney-metabolic syndrome: a narrative review

**DOI:** 10.3389/fendo.2025.1554637

**Published:** 2025-06-05

**Authors:** Yuqing Wang, Yaqing Wang, Xiaojie He, Xiaodong Li

**Affiliations:** ^1^ Graduate School of Hebei Medical University, Shijiazhuang, Hebei, China; ^2^ Graduate School of Chengde Medical University, Chengde, Hebei, China; ^3^ Department of Nephrology, Baoding No.1 Central Hospital of Hebei Medical University, Baoding, Hebei, China

**Keywords:** sodium-glucose transporter 2 inhibitors, comorbidities, cardiovascular system, chronic kidney disease, metabolic syndrome, diabetes

## Abstract

The Cardiovascular-Kidney-Metabolic (CKM) syndrome is a systemic disorder involving obesity, diabetes, chronic kidney disease (CKD), and cardiovascular disease, characterized by complex pathophysiological mechanisms that interact and lead to increased morbidity and mortality. In recent years, sodium-glucose transport protein 2 inhibitors (SGLT2i), as a new class of antidiabetic medications, have shown remarkable efficacy in the management of diabetes, renal and cardiovascular diseases. Research has confirmed their ability to reduce cardiovascular events and all-cause mortality. These inhibitors lower blood glucose levels by decreasing renal reabsorption of glucose and sodium, and offer multiple benefits, including lowering blood pressure, reducing body weight, exerting antioxidant, anti-inflammatory, and anti-fibrotic effects, as well as reducing proteinuria and improving glomerular filtration rate. These effects collectively contribute to the improvement of cardiovascular and renal health. Furthermore, SGLT2i have shown potential therapeutic roles at various stages of CKM syndrome, including improving cardiac function, slowing CKD progression, promoting weight loss, and improving lipid profiles. However, the precise mechanisms of action and off-target effects of SGLT2i still require further investigation to evaluate their efficacy and safety under different clinical conditions. Future research directions should include strategies for multiple disease management, combination therapy effects, interdisciplinary collaboration, and long-term follow-up studies to fully understand and optimize the application of SGLT2i in the treatment of CKM syndrome.

## 1 Introduction

Cardiovascular and renal damage are widespread clinical issues, and metabolic disorders such as diabetes, along with obesity and glucose intolerance in prediabetic states, can lead to chronic damage to the heart and kidneys. In October 2023, the American Heart Association introduced a Presidential Advisory, proposing a novel concept known as Cardiovascular-Kidney-Metabolic Syndrome (CKM). CKM is defined as a systemic condition resulting from the pathophysiological interactions among obesity, diabetes, chronic kidney disease (CKD), and cardiovascular diseases (CVD), including heart failure (HF), atrial fibrillation (AF), coronary artery disease, stroke, and peripheral artery disease (1). This syndrome is characterized by significant associations between cardiovascular, renal, and metabolic diseases. Extensive epidemiological data indicate that the onset of one disease frequently exacerbates others, increasing morbidity and mortality through shared pathophysiological mechanisms. In an observational cohort study involving adults with Diabetes mellitus type 2 (T2DM) who commenced oral hypoglycemic therapy between 2008 and 2018 and had no history of cardiovascular disease or CKD, it was found that approximately 24% of initial events during the observation period were associated with HF, and 36% were related to the progression of CKD (2, 3). Researchers utilized outpatient registry data from U.S. healthcare facilities specializing in primary care, cardiology, and endocrinology to conduct a study assessing the prevalence of cardiovascular, renal, and metabolic diseases, as well as their comorbidities, in adults with T2DM. The study revealed that only 6.4% of patients had T2DM without concurrent renal or metabolic diseases, while about 51% of participants had three or more comorbid conditions, with age and glycemic control potentially contributing to this high prevalence. The most commonly observed conditions included hypertension, dyslipidemia, cardiovascular disease, and CKD, either as single or multiple coexisting conditions (4). The CKM syndrome facilitates the identification and management of diseases that concurrently affect the heart, kidneys, and metabolic systems, emphasizing the need for interdisciplinary collaboration among clinicians to develop comprehensive management strategies addressing various systems. Thus, exploring the interconnections between different systems is vital for formulating effective treatment strategies.

Epidemiological studies highlight the clinical burden of CKM syndrome. For instance, 25%-63% of heart failure patients exhibit coexisting renal dysfunction, while over 50% of individuals with type 2 diabetes present with three or more comorbid conditions, underscoring the urgent need for integrated therapeutic strategies (5).

Recently, SGLT2 inhibitors (SGLT2i) have emerged as a novel class of antidiabetic agents with pleiotropic benefits across multiple organ systems. Beyond glycemic control, these agents reduce cardiovascular mortality and hospitalization for heart failure, slow the progression of chronic kidney disease by modulating renal hemodynamics, and improve metabolic parameters through weight loss and lipid profile optimization. SGLT2i function by inhibiting the sodium-glucose co-transporter 2 in the proximal renal tubule, thereby reducing glucose reabsorption and promoting glycosuria and natriuresis, ultimately lowering blood glucose levels (6, 7). Additionally, SGLT2i have demonstrated the ability to lower blood pressure and reduce body weight, which may have beneficial effects on cardiovascular and renal health. Furthermore, the long-term clinical outcomes of the EMPA-REG OUTCOME trial evaluate that SGLT2i play a significant role through antioxidant, anti-inflammatory, and anti-fibrotic pathways, significantly reducing cardiovascular events and all-cause mortality in patients with T2DM (8, 9).

Given the beneficial effects of SGLT2i in diabetes and cardiovascular disease, investigating their potential application and clinical significance within the context of CKM syndrome is particularly important. Despite existing research indicating the significant therapeutic effects of SGLT2i in cardiovascular, renal, and metabolic diseases, studies specifically addressing their management and application in CKM syndrome remain relatively limited. Therefore, further clinical trials and mechanistic studies are necessary to validate the potential efficacy and safety of SGLT2i in CKM syndrome, thereby providing additional evidence to support clinical practice. This narrative review aims to synthesize current evidence on the therapeutic effects of SGLT2i in CKM syndrome, elucidate their pathophysiological mechanisms across cardiovascular, renal, and metabolic systems, and highlight future research directions to optimize their clinical application.

## 2 Overview of the CKM syndrome

### 2.1 Stages of the CKM syndrome

Current research indicates that CKM, characterized as a progressive disease, typically arises from the pathological expansion and accumulation of excessive and dysfunctional adipose tissue. It often develops into related metabolic diseases such as hypertension, hypertriglyceridemia, T2DM, metabolic syndrome, cardiovascular disease, and renal disease (10). As the disease progresses, it leads to a high risk of clinical cardiovascular disease, renal failure, disability, and death. Therefore, early detection and management of CKM syndrome necessitate screening for relevant risk factors throughout the entire life cycle, identifying stages of CKM syndrome early to enhance prevention and management for the population. The staging of CKM syndrome diagnosis is proposed by the AHA, and it is classified from stage 0 to stage 4 based on individual metabolic risk factors (**Table 1**), the presence of CKD, subclinical cardiovascular disease, and clinical cardiovascular disease (1).

**Table 1:** Staging of Cardiovascular-Kidney-Metabolic syndrome.

Stage	Description	
Stage 0	No cardio-renal metabolic risk factors. Normal BMI and waist circumference, normal blood glucose, blood pressure, and blood lipids. No evidence of CKD or subclinical/clinical CVD.	
Stage 1	Excess/disordered adipose tissue, overweight status or obesity; abdominal obesity leading to impaired glucose tolerance.	
Stage 2	Excess or functionally abnormal adipose tissue. BMI ≥25 kg/m² (≥23 kg/m² for Asian populations). Waist circumference ≥88 cm for women, ≥102 cm for men (≥80/90 cm for Asian women/men). Fasting blood glucose 100–124 mg/dL or HbA1c 38–46 mmol/mol. No other metabolic risk factors or CKD.	
Stage 3	Presence of metabolic risk factors (hypertriglyceridemia, hypertension, diabetes, metabolic syndrome) and/or moderate to high-risk CKD.	
Stage 4	CKM with clinical CVD. Presence of excessive/dysfunctional obesity, other CKM risk factors, or CKD along with clinical CVD (coronary artery disease, heart failure, stroke, peripheral arterial disease, atrial fibrillation).	
	4a	Stage 4 without kidney failure.
	4b	Stage 4 with kidney failure.

CKD chronic kidney disease; CVD cardiovascular diseases; HbA1c Glycated Hemoglobin A1c; BMI is calculated as weight in kilograms divided by height in meters squared (kg/m²).

### 2.2 Pharmacotherapy recommendations for the management of CKM syndrome

In terms of treatment and management strategies, the primary goal for managing early-stage (stages 1-3) CKM is the prevention of CVD. This encompasses weight reduction, blood pressure control, lipid regulation, glucose management, and the management of subclinical CVD and CKD. For patients at stage 4 CKM, more proactive weight management interventions are necessary (pharmacotherapy is recommended for BMI ≥ 27 kg/m²), in conjunction with lipid regulation, blood pressure control, glycemic management, and comprehensive CKD management (1). The following pharmacotherapy recommendations are suggested for the management of CKM syndrome (**Table 2**) (1).

**Table 2:** Treatment and management strategies for Cardiovascular-Kidney-Metabolic syndrome.

CKm Stage	Recommendations
CKM 1	Weight management: Consider pharmacotherapy when BMI ≥ 30 kg/m²
CKM 2	Elect evidence-based medications based on risk factors: Antihypertensives: ACEi/ARB; Lipid management: statins, fibrates, EPA; Diabetes management: SGLT2i, GLP-1RA, metformin; CKD management: ACEi/ARB, SGLT2i, etc;
CKM 3	Managing subclinical cardiovascular disease: statins, PCSK9i, aspirin, GLP-1RA, IPE, ACEi/ARB, β-blockers, SGLT2i;
CKM 4	1.Aggressive weight management: Consider pharmacotherapy when BMI≥27 kg/m²; 2.Comprehensive management: lipid regulation, blood pressure control, glycemic management, CKD management; 3.Diabetic patients, prioritize SGLT2i orGLP-1RA to reduce the risk of major adverse cardiovascular events (MACE). 4.Patients with concurrent CKD and heart failure, prioritize SGLT2i. 5.Individuals with HbA1c ≥ 9%, high insulin doses, and BMI ≥ 35 kg/m², prioritize GLP-1RA. 6.Patients with multiple CVD may benefit from the combined use of SGLT2i and GLP-1RA.

ACEI Angiotensin-Converting Enzyme inhibitors; ARB Angiotensin II Receptor Blockers; EPA Eicosapentaenoic Acid; GLP-1RA Glucagon-Like Peptide-1 Receptor Agonists; PCSK9i Proprotein Convertase Subtilisin/Kexin Type 9 Inhibitors; IPE Inositol Phosphoglycan Eicosanoid; CVD cardiovascular diseases.

## 3 Pathophysiological mechanisms of CKM syndrome

**Figure 1:**
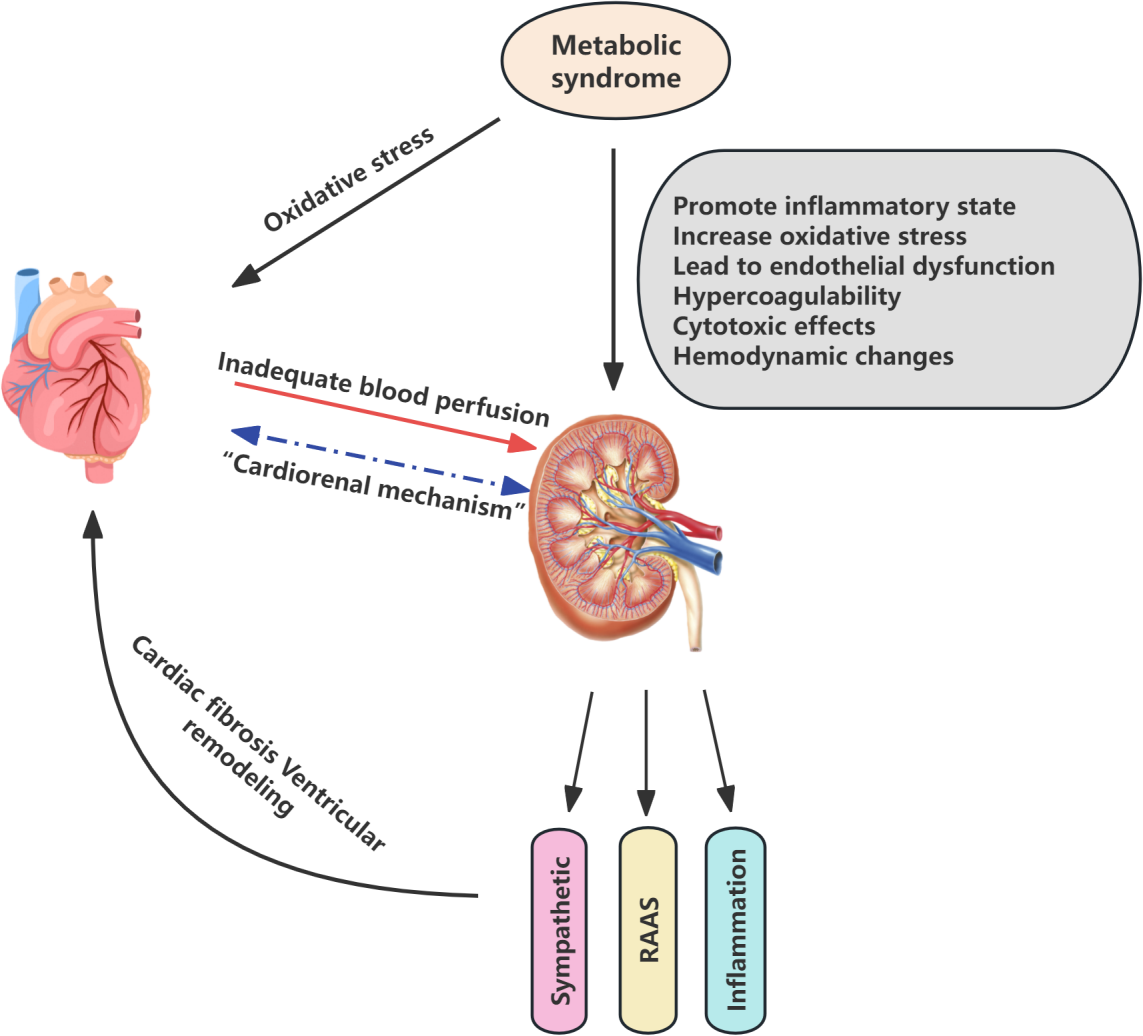
Pathophysiological mechanisms of Cardiovascular-Kidney-Metabolic syndrome. RAAS renin-angiotensin-aldosterone system.

The pathophysiological mechanisms underlying CKM syndrome are intricate and involve numerous interrelated biological processes. Cardiovascular, renal, and metabolic diseases interact at the pathophysiological level, exhibiting shared underlying mechanisms, the activation of which initiates a vicious cycle disease progression and contributes to rising morbidity and mortality (11). HF, as a leading cause of cardiovascular mortality, has increased in prevalence in recent years, paralleled by a growing trend in renal insufficiency. The interaction between the heart and kidneys, known as cardiorenal mechanisms, plays a pivotal role in the evolution of CKM syndrome. Cardiac dysfunction may lead to inadequate renal perfusion, thereby provoking or exacerbating renal injury; similarly, renal impairment can affect cardiac structure and function through multiple pathways, such as the activation of the renin-angiotensin-aldosterone system (RAAS), sympathetic nervous system activation, and enhancement of inflammatory and oxidative stress responses. These processes result in cardiac fibrosis and ventricular remodeling, further burdening the heart and ultimately leading to a state of mutual dependence and vicious cycle, forming the cardiorenal syndrome (12–14). Moreover, metabolic syndrome elevates the risk of CVD through inflammation, oxidative stress, and associated metabolic alterations (15). In a similar vein, metabolic syndrome affects the kidneys through a multitude of mechanisms, including the promotion of an inflammatory state, elevation of oxidative stress levels, induction of endothelial dysfunction, creation of a prothrombotic state, as well as the induction of direct cytotoxic effects and alterations in hemodynamics. These factors collectively contribute to the early onset, accelerated progression, and deterioration of kidney disease (16). Thus, the interactions among the cardiac, renal, and metabolic systems create a complex network of interactivity (**Figure 1**).

### 3.1 The effects of SGLT2i on the cardiovascular system

#### 3.1.1 Integrated mechanisms of cardiovascular protection

**Figure 2:**
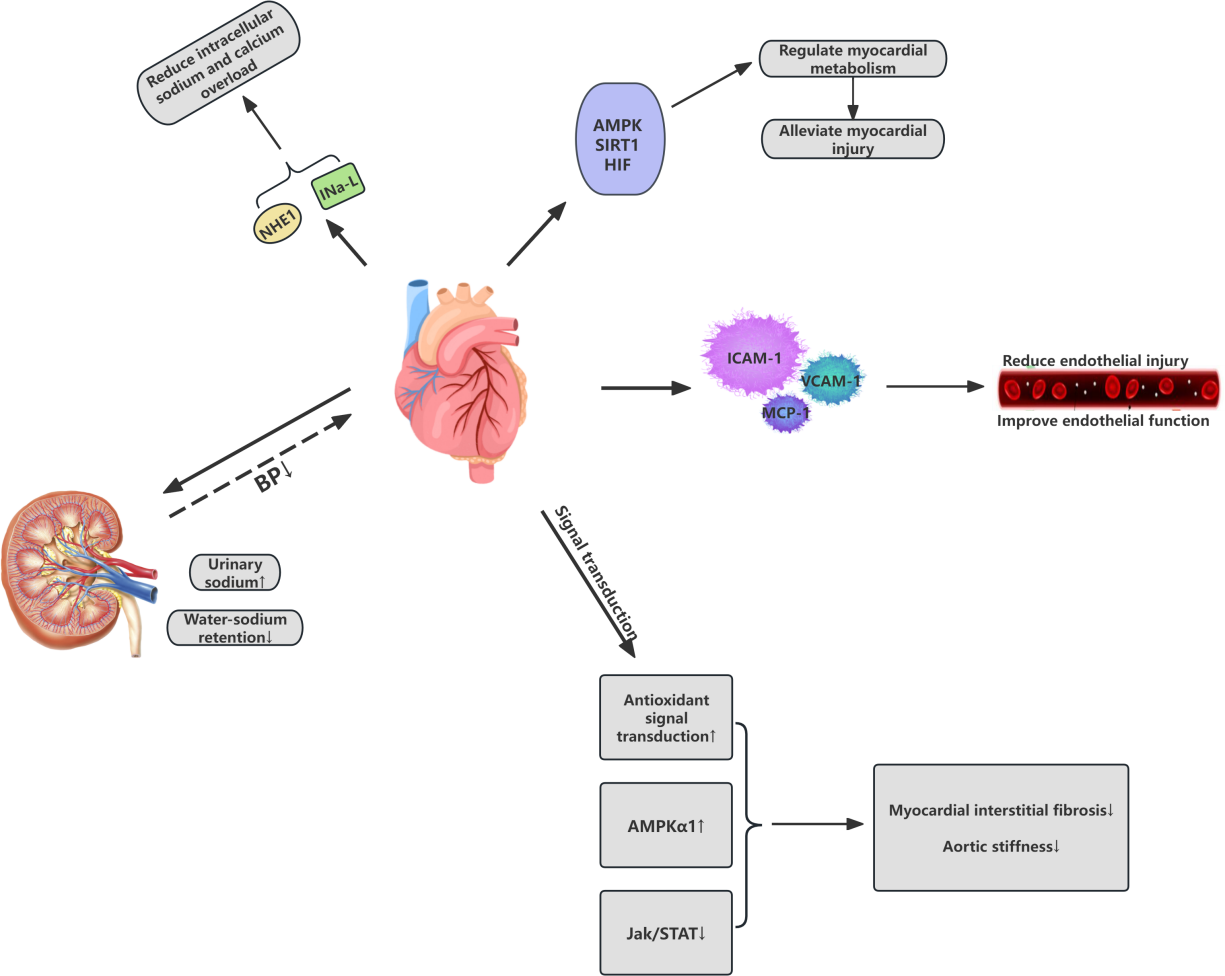
Cardiovascular effects of SGLT2 inhibitors in Cardiovascular-Kidney-Metabolic syndrome. IC Ca, Intracellular calcium; NE, Norepinephrine; NHE1 cardiac sodium-hydrogen exchanger 1; AMPK AMP-Activated Protein Kinase; SIRT1 Sirtuin 1; HIF Hypoxia-Inducible Factor; BP Blood Pressure.

The cardiovascular protective effects of SGLT-2 inhibitors in patients may involve various mechanisms, including the reduction of both preload and afterload on the heart, optimization of myocardial energy metabolism, inhibition of abnormal cardiac remodeling, delay of atherosclerosis, and improvement of multiple cardiovascular risk factors. SGLT-2 inhibitors facilitate the excretion of sodium through the urine, thereby reducing sodium and fluid retention, leading to a modest reduction in blood pressure. Additionally, these inhibitors contribute to a reduction in energy balance, which indirectly lowers body weight, and help alleviate cardiac workload, reduce mortality from cardiovascular events, and decrease hospitalization rates for HF. These effects are significant for individuals regardless of their cardiovascular disease history (17). Furthermore, SGLT-2 inhibitors have been shown to mitigate endothelial damage and improve endothelial function by decreasing the expression of inflammatory molecules such as adhesion molecules and macrophage markers. Under the influence of SGLT-2 inhibition, ventricular myocardial interstitial fibrosis is reduced through antioxidant signaling, activation of AMP-activated protein kinase, and attenuation of the Jak/STAT signaling pathway, leading to improvements in aortic stiffness and overall cardiac function (18–20). The following diagram summarizes the mechanism of action of SGLT2 inhibitors on the cardiovascular system (**Figure 2**).

Notably, recent studies have also indicated that, in addition to their remarkable cardiovascular protective effects, SGLT-2 inhibitors may hold potential in the treatment of arrhythmias.

#### 3.1.2 Potential role in anti-arrhythmia therapy

According to an analysis of the global combined electronic health record database, studies have demonstrated that various SGLT2i can reduce the incidence of AF and all-cause mortality, while also decreasing the overall risk of ventricular tachycardia/ventricular fibrillation and cardiac arrest (21). Clinical trials have confirmed the application of SGLT2i in reducing ventricular arrhythmias. For instance, data from the DAPA-HF study revealed that dapagliflozin significantly reduces the risk of severe ventricular arrhythmias, cardiac arrest, and sudden death in patients with HF (22). Additionally, SGLT2i play a crucial potential role in reducing the recurrence risk of AF and improving the prognosis of AF patients. A prospective, randomized controlled trial showed that tofogliflozin, an SGLT2 inhibitor, was more effective than the dipeptidyl peptidase-4 inhibitor anagliptin in reducing the recurrence of AF in patients with T2DM (23).

SGLT2i may provide direct cardioprotective effects against arrhythmias by modulating the cardiac sodium-hydrogen exchanger 1 and the late sodium current (INa,L), thereby reducing intracellular sodium and calcium overload (24, 25). Additionally, these inhibitors might indirectly impact the cardiovascular system through their influence on myocardial metabolism and autophagic processes. SGLT2i are reported to activate molecular pathways, including AMPK, SIRT1, and HIF, thereby triggering a transcriptional response similar to conditions of nutrient and oxygen deprivation, which in turn modulates myocardial metabolism (26). These molecular mediators coordinately regulate autophagy—a conserved lysosomal degradation mechanism essential for maintaining cellular homeostasis through selective clearance of impaired organelles. This regulatory cascade simultaneously suppresses NLRP3 inflammasome activation, thereby mitigating cardiomyocyte metabolic dysfunction and coronary microvascular endothelial damage (26, 27). The synergistic modulation of these pathophysiological processes ultimately confers cardioprotective effects through secondary mechanisms.

In conclusion, SGLT2i show significant promise in cardiovascular therapy, improving glycemic control and potentially reducing the risk of cardiovascular events. Clinical trials have demonstrated their benefits in HF, myocardial infarction, and arrhythmias. However, the underlying mechanisms and off-target effects require further investigation to fully evaluate their efficacy and safety across various clinical settings.

### 3.2 Renal effects of SGLT2 inhibitors

SGLT2i reduce blood glucose levels by inhibiting the activity of SGLT2 in the proximal convoluted tubules, thereby decreasing renal reabsorption of sodium and glucose. Landmark trials such as DAPA-CKD and EMPA-KIDNEY have further validated the renoprotective effects of SGLT2i. In DAPA-CKD, dapagliflozin reduced the composite risk of sustained ≥50% eGFR decline, end-stage kidney disease, or renal/cardiovascular death by 39% in patients with CKD, regardless of diabetes status. Similarly, EMPA-KIDNEY demonstrated that empagliflozin significantly lowered the risk of kidney disease progression or cardiovascular death in a broad CKD population, reinforcing the class effect of SGLT2i in renal protection (28–30). In recent years, significant progress has been made in the study of SGLT2i renal protective effects, particularly in patients with CKD and T2DM, where they have shown notable renal benefits.

**Figure 3:**
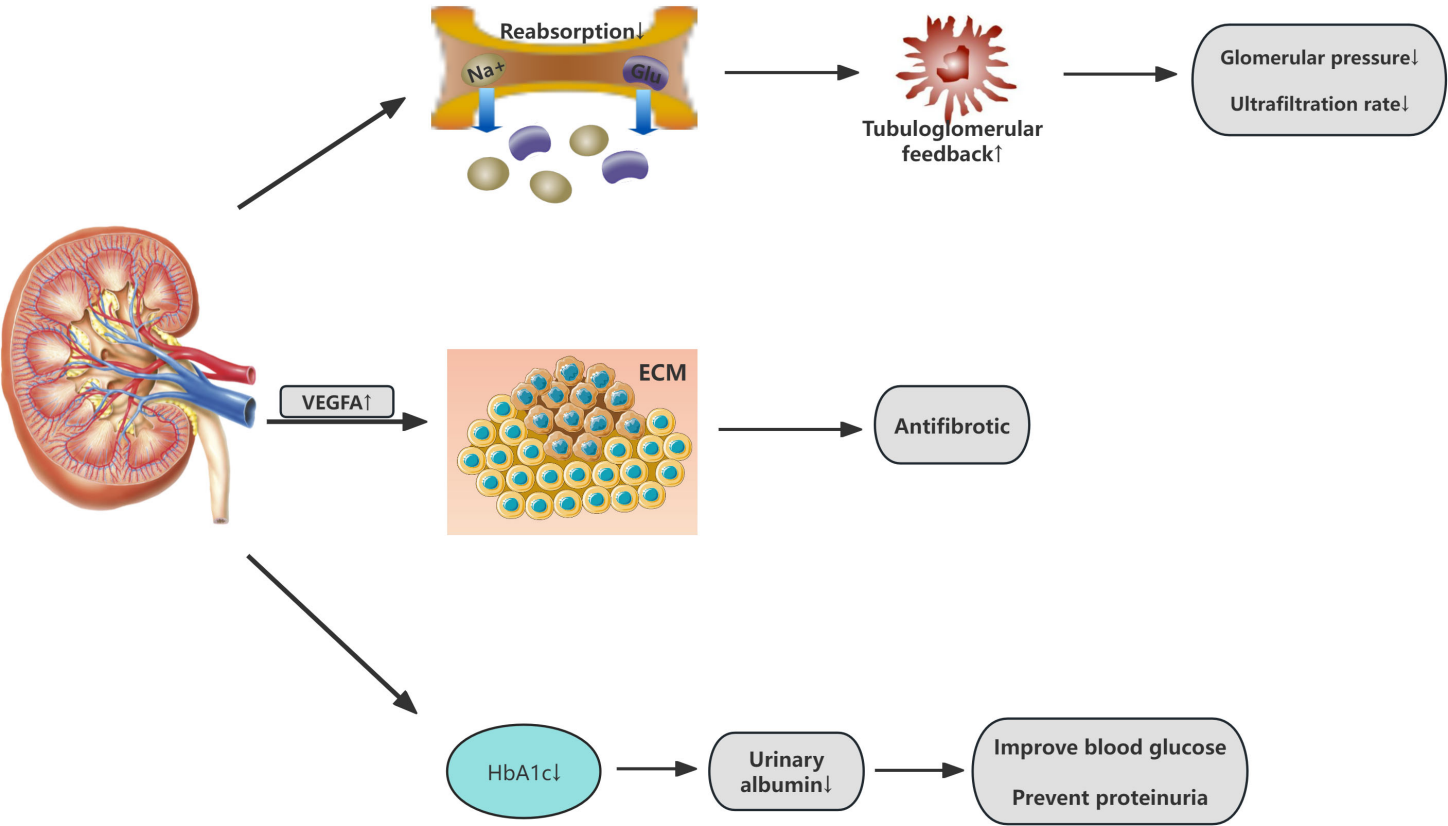
Renal effects of SGLT2 inhibitors in Cardiovascular-Kidney-Metabolic syndrome. VEGFA vascular endothelial growth factor A; ECM Extracellular Matrix.

SGLT2i can slow the progression of CKD, reducing the risk of kidney failure, elevated serum creatinine, and other renal events. Notably, the reduction in albuminuria and preservation of GFR by SGLT2i are not secondary to glycemic control but are mediated through direct renal mechanisms. These include restoration of glomerular hemodynamics, attenuation of hyperfiltration, and suppression of pro-fibrotic signaling pathways (e.g., TGF-β and NF-κB) (30–32). However, research by Heerspink et al. suggests that canagliflozin may have a kidney-protective effect independent of its glycemic action (33). primarily attributed to alterations in renal hemodynamics. By reducing the reabsorption of sodium and glucose in the proximal tubule, SGLT2i restore the abnormal glomerular-tubular feedback mechanism, inducing vasoconstriction of the afferent arteriole, lowering glomerular pressure, and decreasing hyperfiltration (34), thus providing renal protection from a hemodynamic perspective. Furthermore, studies indicate that SGLT2i may exert antifibrotic effects through various mechanisms, including the modulation of extracellular matrix metabolism, regulation of anti-inflammatory and antioxidative pathways, and enhancement of vascular endothelial growth factor A (VEGFA) expression (35, 36). thereby slowing the progression of kidney disease. Additionally, SGLT2i possess multifaceted actions such as weight reduction, diuresis, blood pressure lowering, and uric acid reduction, which may also indirectly protect the kidneys. For example, by inducing osmotic diuresis, they reduce both cardiac preload and afterload, which alleviates interstitial fluid accumulation and decreases edema. This, in turn, provides indirect renal protection (37). The following diagram illustrates the mechanism of action of SGLT2 inhibitors on the renal system (**Figure 3**).

### 3.3 The improvement of SGLT2 on metabolic diseases

Increasing clinical research demonstrates that, compared to medications that solely improve glucolipid metabolism and blood pressure, SGLT2i exhibit a multi-target effect that underscores their advantages in treating metabolic diseases.

#### 3.3.1 SGLT2i curb obesity and reduce body weight

Over the past fifty years, the global incidence of obesity has increased significantly, reaching epidemic levels. Due to its correlation with heightened risks of multiple diseases, alongside significant reductions in both quality of life and life expectancy, obesity has emerged as a significant health challenge (38). Clinical studies have shown that the primary mechanism for early weight reduction through SGLT2i is the promotion of osmotic diuresis leading to a decrease in volume (39). However, a reduction in body water stimulates the secretion of antidiuretic hormone, promoting the reabsorption of water, and thus, levels stabilize after four weeks. Consequently, the long-term weight loss effects of SGLT2i are related to their impact on fat metabolism. SGLT2i can alter the form of energy provision, facilitating a metabolic shift from carbohydrate to lipid metabolism and promoting the browning of white adipose tissue. Brown adipose tissue, a principal source of heat production under cold conditions and stress, has been shown to combat obesity (40). SGLT2i facilitate a shift from carbohydrate metabolism to fatty acid and ketone metabolism, increasing the glucagon-to-insulin ratio and fat utilization rates, thereby reducing body weight (41). Furthermore, in murine studies, SGLT2i can degrade fat through a series of physiological and biochemical processes within the liver-brain-adipose neurocircuitry, and by upregulating genes associated with β-oxidation in liver cells, they downregulate genes related to lipid synthesis, promoting the oxidation of fatty acids in the liver and reducing lipid synthesis (42, 43).

#### 3.3.2 SGLT2i ameliorate hyperglycemia

SGLT2i enhance glycemic control through the reduction of glucose reabsorption and the facilitation of its excretion, effectively promoting insulin clearance and alleviating hyperinsulinemia to improve insulin resistance (44). Insufficient insulin secretion and resistance are primary factors in abnormal glucose elevation, thus exploring mechanisms to mitigate insulin resistance and stabilize blood glucose levels is crucial for managing metabolic dysregulation. Studies have revealed that transepithelial glucose absorption is an active process dependent on Na+/K+ ATPase, driven by a sodium gradient across the brush border membrane (45). with SGLT2i reducing glucose transport by inhibiting SGLT2. Additionally, these inhibitors improve the structure and function of adipose tissue, alleviating obesity-related insulin resistance. Research has shown that SGLT2i can enhance the differentiation of epicardial adipose tissue, reduce the secretion of pro-inflammatory chemokines and cytokines, thus decreasing epicardial fat deposition and enhancing lipid utilization to relieve insulin resistance (46, 47). However, studies involving dapagliflozin on pancreatic cells *in vitro* and *in vivo* indicate that by reducing blood glucose, dapagliflozin indirectly affects pancreatic cell function through a temporary increase in glucagon secretion and a reduction in insulin secretion (48). suggesting that the role of SGLT2i in improving insulin resistance and its associated effects warrants further investigation.

#### 3.3.3 SGLT2i reduce hypertension

In line with the World Health Organization’s global prevention objectives, hypertension plays a critical role in exacerbating renal and cardiovascular complications among patients with CKD, which not only leads to deteriorating renal function but also intensifies the severity of hypertension itself (49). Thus, exploring the mechanisms by which SGLT2i reduce hypertension is also crucial. Studies have found that SGLT2i are likely linked with the suppression of the sympathetic nervous system, significantly reducing the levels of tyrosine hydroxylase and norepinephrine (50). diminishing the sympathetic nervous system’s impact on effector organs, thereby lowering blood pressure. Additionally, SGLT2i enhance the release of nitric oxide (NO), a vasodilation-promoting agent from vascular endothelium. NO production depends on the activity of endothelial nitric oxide synthase (eNOS). Recent studies have discovered that engliflozin and dapagliflozin, by promoting eNOS phosphorylation and enhancing NO production, reduce oxidative stress and inflammatory responses, thereby lowering blood pressure and increasing the bioavailability of endothelial NO, collectively reducing hypertension (51, 52). Moreover, SGLT2i also contribute to osmotic diuresis and natriuresis, reducing blood volume and thereby lowering blood pressure.

#### 3.3.4 SGLT2i ameliorate lipid metabolism disorders

The characteristics of diabetic dyslipidemia include elevated serum triglycerides (TG), reduced high-density lipoprotein cholesterol (HDL-C), and increased levels of small dense low-density lipoproteins (sLDL). An increasing body of clinical meta-analyses and studies has confirmed that SGLT2i can modestly increase HDL-C and decrease TG, thereby ameliorating lipid metabolism disorders in diabetic patients and reducing the risk of CVD (53). Although some studies suggest that SGLT2i may elevate low-density lipoprotein cholesterol(LDL-C)levels, this mechanism is likely associated with reduced expression and activity of LDL receptors (54).However, there is controversy regarding changes in LDL-C levels; the small, dense LDL-C subtype is more likely to contribute to atherosclerosis, while the large, buoyant LDL-C subtype has a relatively weaker impact. Research indicates that the LDL-C increase associated with SGLT2i predominantly involves the large, buoyant LDL components, which are less likely to elevate cardiovascular disease risk (55, 56). In summary, the effects of SGLT2i in lowering triglycerides and elevating HDL-C may serve as potential protective factors in preventing atherosclerotic cardiovascular disease.

**Figure 4:**
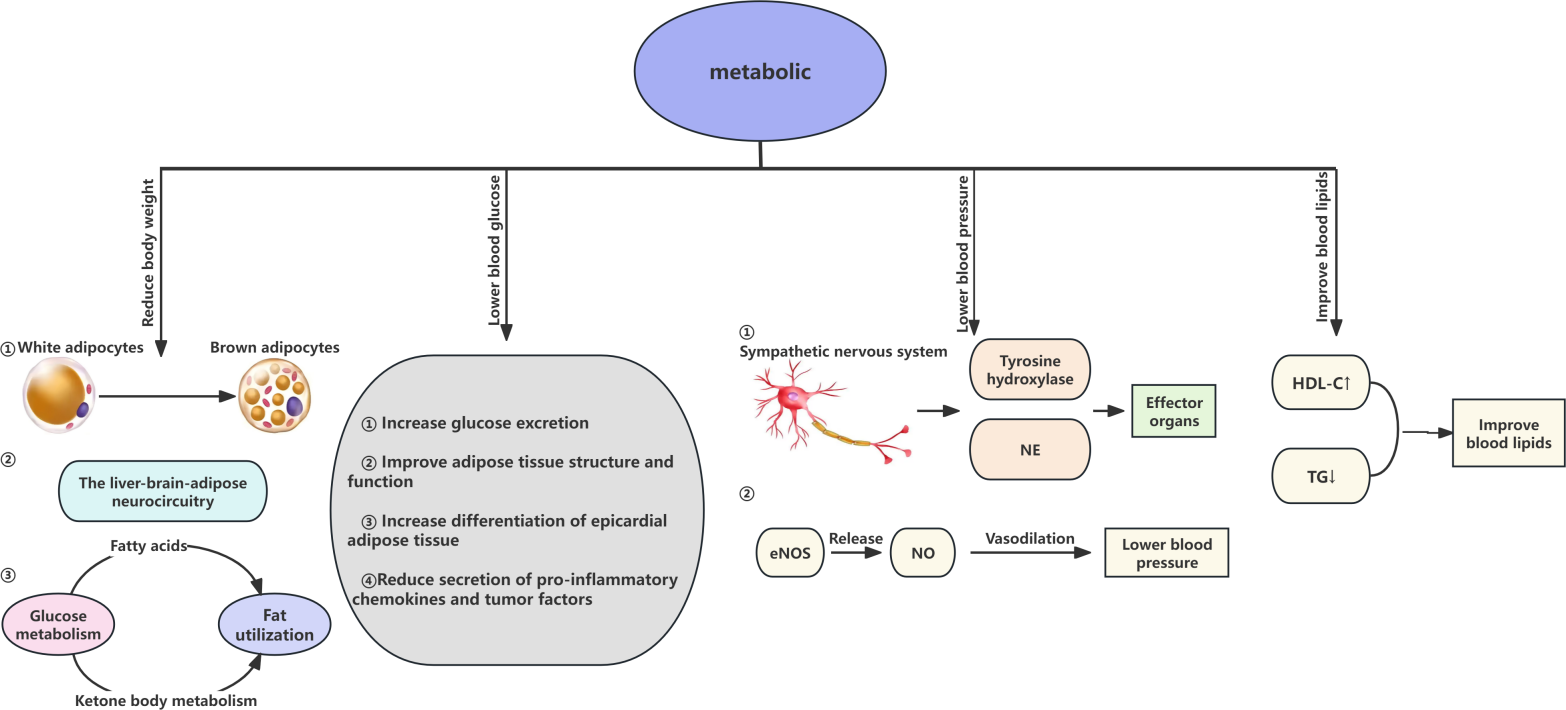
Renal effects of SGLT2 inhibitors in Cardiovascular-Kidney-Metabolic syndrome. NE norepinephrine; eNOs endothelial nitric oxide synthase; NO nitric oxide; HDL-C High-Density Lipoprotein Cholesterol; TG Triglycerides.

From the above mentioned, SGLT2i have shown potential in treating metabolic diseases due to their effects in reducing body weight, lowering lipid levels, and decreasing blood pressure while also providing protective benefits to the cardiovascular and renal systems (**Figure 4**).

## 4 Safety considerations

Despite their proven efficacy, SGLT2i are associated with specific adverse effects that require clinical vigilance. Common risks include genitourinary infections due to glycosuria, volume depletion-related events, and a potential increase in LDL-C levels. Rare but severe complications such as euglycemic diabetic ketoacidosis and Fournier’s gangrene have been reported, particularly in high-risk populations (57). A recent meta-analysis has indicated that these risks are generally manageable through patient selection, dose adjustment, and routine monitoring (58).

## 5 Conclusion and prospect

SGLT2i exhibit multifaceted therapeutic benefits in CKM syndrome, spanning cardiovascular, renal, and metabolic systems. Their ability to reduce cardiovascular mortality, slow CKD progression, and improve metabolic dysregulation is mediated through hemodynamic optimization, anti-inflammatory/antifibrotic effects, and metabolic reprogramming. Clinical trials such as EMPA-REG OUTCOME, DAPA-CKD, and EMPA-KIDNEY have robustly validated these effects across diverse patient populations, including those without diabetes (59–61). Moreover, research findings indicate that, regardless of diabetes status, participants experience reductions in cardiovascular mortality, heart failure hospitalization rates, and the incidence of renal failure through antioxidant, anti-inflammatory, and anti-fibrotic pathways, providing a theoretical foundation for their application in CKM syndrome. Throughout different stages of CKM syndrome, SGLT2i demonstrate potential therapeutic effects, including improving cardiac function, slowing CKD progression, and improving lipid profiles.

However, further research is needed to understand the precise mechanisms and non-target effects of SGLT2i to evaluate their efficacy and safety in various clinical settings. Future research on SGLT2i in CKM syndrome should enhance multi-disease management strategies to explore their comprehensive application, particularly their effects on improving cardiovascular, renal, and metabolic health. The effectiveness of combining SGLT2i with other medications, such as RAAS inhibitors and GLP-1 receptor agonists, should be examined to achieve comprehensive management of patients with CKM syndrome. Interdisciplinary cooperation among specialists in cardiology, nephrology, endocrinology, and molecular biology is encouraged to deepen understanding and address the complexities of CKM syndrome. Long-term follow-up studies are also essential to assess the long-term outcomes of SGLT2i in patients with CKM syndrome, including cardiovascular events, renal failure, and mortality rates.

Additionally, although the overall safety profile of SGLT2i is favorable, potential side effects, such as risks of genitourinary infections, hypotension, ketoacidosis, and fractures, require close monitoring and management in clinical practice. By further optimizing treatment strategies and enhancing understanding of the mechanisms of action of SGLT2i, their potential in treating CKM syndrome can be fully realized, offering more effective and safer therapeutic options for patients.
